# A Three-Axis Force Sensor for Dual Finger Haptic Interfaces

**DOI:** 10.3390/s121013598

**Published:** 2012-10-10

**Authors:** Marco Fontana, Simone Marcheschi, Fabio Salsedo, Massimo Bergamasco

**Affiliations:** PERCRO Laboratory, TeCIP Institute, Scuola Superiore Sant'Anna, Piazza Martiri della Libertà 33, Pisa 56127, Italy; E-Mails: s.marcheschi@sssup.it (S.M.); f.salsedo@sssup.it (F.S.); m.bergamasco@sssup.it (M.B.)

**Keywords:** force sensor, Maltese cross, three-axis force sensing, haptics, force feedback, haptic interface

## Abstract

In this work we present the design process, the characterization and testing of a novel three-axis mechanical force sensor. This sensor is optimized for use in closed-loop force control of haptic devices with three degrees of freedom. In particular the sensor has been conceived for integration with a dual finger haptic interface that aims at simulating forces that occur during grasping and surface exploration. The sensing spring structure has been purposely designed in order to match force and layout specifications for the application. In this paper the design of the sensor is presented, starting from an analytic model that describes the characteristic matrix of the sensor. A procedure for designing an optimal overload protection mechanism is proposed. In the last part of the paper the authors describe the experimental characterization and the integrated test on a haptic hand exoskeleton showing the improvements in the controller performances provided by the inclusion of the force sensor.

## 1. Introduction

Force sensors are effectively employed in several contemporary technology application fields. Examples of applications of force sensors can be found in the fields of aerospace and the automotive industry, industrial machinery, robotics and automation, civil and construction engineering, glass, iron and steel, power plants, metallurgy, mining, oil and gas, paper, vibration detection, seismology and several other fields. Reviews of force sensors and their applications can be found in [1,2].

There exist several basic principles for the realization of the transduction of force into electrical signals. Among them mechanical sensors based on strain gauges are among the most widespread and well established. Many commercial products are available on the market, including load cells with single and multi-axis sensing. Moreover researchers have conceived several optimized mechanical designs for the implementation of three-axis [3–6], four-axis [7] and six-axis [8–10] force sensors.

The authors of this paper propose the design of a force sensor that is specifically conceived for kinesthetic haptic interfaces (henceforth called “haptic interfaces” or “haptics”). Kinesthetic haptic interfaces can be functionally seen as robotic manipulators with specific characteristics and performances such as large force bandwidth, high force and position resolution. From the point of view of their mechanical design such specific performances impose some particular requirements such as low inertia, low friction (especially static friction), low encumbrances, light weight of the components and high force-to-weight ratios. The role of force sensors in such devices is to improve their mechanical performance through active compensation of undesired forces. Such a goal can be achieved by exploiting the signal of a force sensor to compensate in real-time any disturbances that affect the output force. From the technical point of view the problem is very similar to the force control of an industrial robotic manipulator, but in the case of haptic interfaces the maximum force requirements are usually lower, and requirements in terms of accuracy and resolution of force exertion are much more demanding. In the field of industrial robotic grippers, sensors are employed to improve the behavior of the robot when a force is applied to the environment. The typical solution consists in sensorizing the end-effector of the robot ensuring that the measured force is the best estimation of the force that is actually exerted by the robot.

In the field of haptic interfaces the integration of force sensors is a common solution to improve mechanical performance and force accuracy [11]. Several haptic devices include commercial force/torque sensors mounted on the end-effector of the device. Haptic interfaces equipped with force sensors have been developed in research laboratories by Frisoli *et al.* [12], Borro *et al.* [13] and Endo *et al.* [14] but they also can be found in commercial haptic devices like the Haptic Master [15]. In such systems the force sensor is integrated in the form of a handle or a tool-like end-effector and the interaction between user and device takes place through an intermediate object replica like a pen or a stylus. On the other hand, only a few works have considered the integration of a force sensor for direct fingertip interaction. Bergamasco *et al.* in [16] developed a custom decoupled force sensor conceived for the integration with the end-effector of a desktop force-feedback device. Ferre *et al.* [17] developed a low-cost fingertip force sensor that employs a pressure sensitive sensor. This last device presents many features suitable for its integration with haptic interfaces such as light weight and low-cost, however its performances in terms of accuracy and hysteresis are quite weak.

In this paper the authors present a study on a mechanical fingertip force sensor aimed at greatly improving the force accuracy of a class of haptic devices. Thus the target is to obtain a very high quality force signal in terms of accuracy, resolution and repeatability, in order to improve resolution and accuracy of the whole haptic device. The proposed design consists of a sensorized thimble that is optimized for the integration with the dual finger haptic exoskeleton described in [18]; however the shape and the global layout are studied to permit the integration of such sensor with other 3-Degrees of Freedom (DoFs) haptic interfaces, as long as they can be equipped with a thimble-like end-effector. The final application that is envisaged is the simulation of the interaction through manipulation of virtual objects using two fingers: thumb and index finger.

In the second section of this paper, the authors provide a brief analysis of the application and define the general architecture for the force sensor. In the third section an analytical model for mechanical dimensioning of the device is presented. In the fourth section the authors propose a criterion for the design and the dimensioning of a mechanism for overload protection of the sensor spring. In the fifth section the dimensioning of a prototype of the sensor is presented along with the experimental characterization. The last section describes the integration with the haptic hand exoskeleton and an experimental validation of the performances of the whole system through the implementation of a closed-loop control method.

## 2. Analysis of the Application

### Dual Finger Haptic Interaction

**Figure 1.**
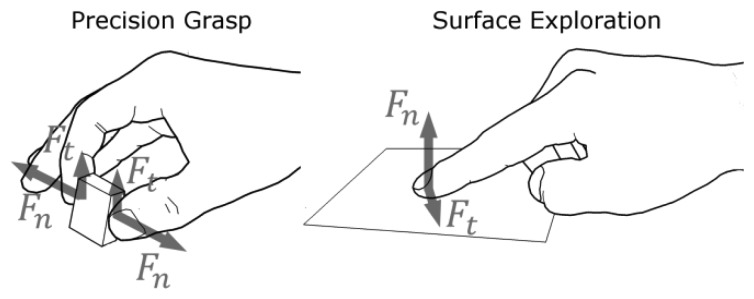
Illustration of the two types of direct finger interaction that are considered for the development of the force sensor. Precision Grasp refers to the grasping of small objects between thumb and index finger while Surface Exploration refers to the stroking of the finger on a surface to explore its qualities.

The artificial generation of the forces that are felt when interacting with objects directly using the hands is the final objective of the haptic hand exoskeleton that has been developed and described in [18]. In particular the target application is the simulation of two types of interaction: (1) Precision Grasp and (2) Surface Exploration (see Figure 1). Jones *et al.* in [19] describe Precision Grasp as the action that is done while holding and manipulating an object between the fingertips of thumb and index finger. Surface Exploration is the common action of exploring the surface of an object by stroking different parts with our hands. Both Precision Grasp and Surface Exploration are extremely complex kinds of hand-object interactions.

Haptic interfaces that are able to replicate such types of interaction must be able to exert forces on both the index and thumb of the user and consequently such kind of devices is usually equipped with thimble-like attachments able to exert forces on the distal phalanxes of the fingers.

In reality, Precision Grasp and Surface Exploration involve contact forces whose intensity varies in a very large range, from tens of Newtons of maximum force that can be exerted (during hard grip) [20] to forces in the order of milli-Newtons for the minimum perceivable force when the finger is passively stimulated [21].

However, while very low forces (in the range of milli-Newtons) are quite frequent and important in everyday manipulative actions, the exertion of maximum forces is a rare event. Analysis of force and their statistical distribution in some common Activities of Day Living (ADL) is provided by Redmond in [22]. These authors show that many small lightweight objects like pens, objects on the desk, portable phones, PCs and phone keyboards, *etc.* are effectively manipulated with forces in the range from fractions of Newtons up to some Newtons.

For this reason, many haptic devices have been designed to operate in the force-range of a few Newtons and accordingly, the target force capability for the presented force sensor has been set in the same range. In particular a maximum operating force of *F_N_* = 5 N has been chosen. At the same time the aim of the design of the proposed force sensor has been oriented toward the optimization of resolution performances in such a range of forces.

**Figure 2.**
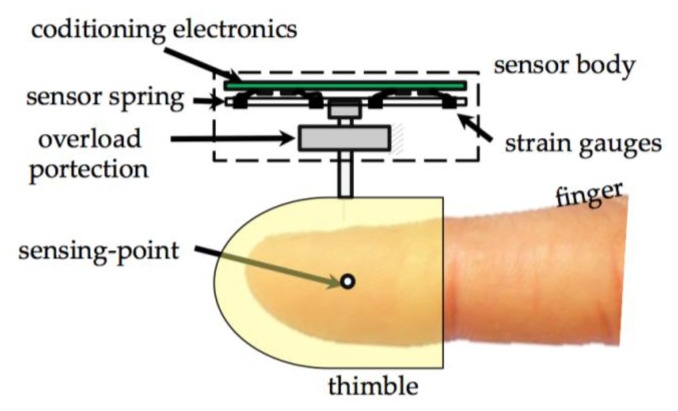
Layout and location of the force sensor respect to the user finger and scheme of internal structure of the components of the force sensor.

Another important issue that strongly conditions the design choices was the constraint raised by the dual finger interaction. The simultaneous interaction with objects using index and thumb finger imposes several limitations for the allowed encumbrances for the physical shape of the force sensor. In particular, to permit the simulation of the grasping of thin objects the encumbrances on the volar side of the finger must be limited as much as possible. Thus, the global shape of the sensor system has been designed according to a layered structure as shown in Figure 2. In this scheme, the force sensor spring and the conditioning electronics have both a flat shape and are disposed over two layers on the dorsal side of the fingertip. The finger is attached to the force sensor through a thimble. It's important to underline that the sensor is connected to the haptic device through a spherical wrist centred on the sensing-point (see Figure 2), thus the force application point is fixed with respect to the fingertip.

## 3. Sensor Design

**Figure 3.**
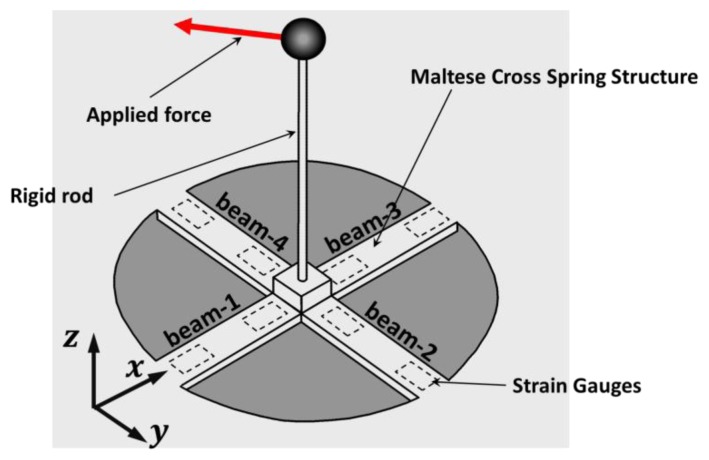
Scheme of the global shape of the adopted spring structure based on a Maltese cross shape.

On the base of the considerations presented in the previous section, the Maltese cross spring shape was selected (see Figure 3). Actually, Maltese cross force sensors are generally force sensors whose spring is cross-shaped. The sensing spring consists of four straight flexible beams whose axis lays on a plane and converge on a rigid central plate (sensing-plate). A rigid rod, whose axis is orthogonal to the plane of the springs, is connected to the central plate. The force is exerted on the tip of the rod and through this element a deformation is induced on the beams. In the developed sensor the rod is also equipped with a thimble as represented in Figure 2.

The Maltese cross is a well-known shape for the implementation of multi-axis force sensors. One of the first examples of 6-DoF force sensors employing this structure is described in [23]. Since then, it has been mainly employed in the field of automation and robotics for end-effector sensorization. Other examples of 6-DoF force sensors employing slightly modified versions of the Maltese-cross can be found in [24–26]. In the field of automotive research another sensor, based on the same architecture, has been developed for the force/torque sensing of loads on a racing tyre [27]. The Maltese cross shape of such designs is optimized for sensing six degrees of freedom. Flexible beams have usually squared cross-section and strain gauges are located on different faces of the beam. This is necessary to obtain the six-axis of sensing but leads to high manufacturing costs, a heavy structure and complex assembly and gluing of gauges.

Three-axis force sensors based on Maltese cross structures have been also developed in the field of robotic-hand design. Researchers have designed and developed miniature three-axis silicone-based force sensors with Maltese-cross shapes. Beccai *et al.* [28] and Vasarhelyi *et al.* [29] developed two similar structures for the implementation of miniature force sensors to obtain a very thin layer sensing skin for robotic fingers. The sensors have been developed and tested, but not integrated on a robotic system.

The authors of the present paper analyze a particular Maltese cross spring that has a flat shape according to the specifications described in the previous section. In this case the strain gauges are disposed on the top and bottom surfaces of flat beams (see Figure 3). This solution allows one to: (1) simplify the assembly process since gluing is done on the faces of a flat planar surface; (2) reduce manufacturing costs since the spring can be obtained by cutting a thin plate of steel or aluminum; (3) reduce weight and assume flat encumbrances according to haptic interface requirements (see Section 2). In the following section we provide an analytical description of the sensing spring assuming the approximation of slender beam theory.

### Characteristic Matrix

A preliminary analytical approach has been adopted for the analysis of the sensor spring through a set of simplification of the physical problem based on the following assumptions and considerations:

- The elastic deformable beams can be approximately treated as slender beams;
- The system has four geometrical symmetry planes through the *z*-axis;
- Deformations occur only in elastic ranges.

**Figure 4.**
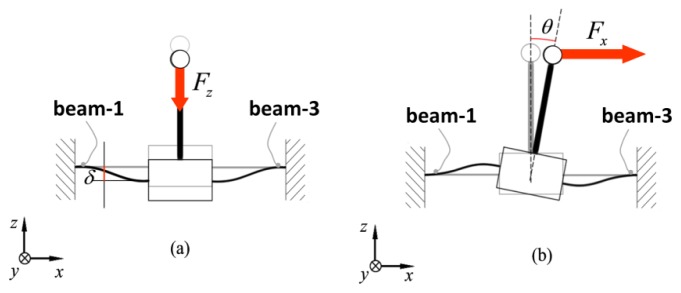
Possible load condition for the Maltese cross force-sensor: Condition A on the left (**a**) and Condition B on the right (**b**).

The spring structure presents several symmetry planes: the *x-z* and *y-z* planes and also the two planes through the *z*-axis that form angles of *π*/4 and 3*π*/4 with the *x*-direction. If strain gauges are also arranged according to those symmetries, there are only two relevant load conditions: the Load Condition A that occurs when force is applied in the *z*-direction [see Figure 4(a)] and Load Condition B that occurs when force is applied in the *x*-direction [see Figure 4(b)]. All the other load conditions derive from the possible combinations of these vertical (Load Condition A) and tangential (Load Condition B) loads.

In the following section the beams are considered as slender-beam and the symbols assume the following meanings: *l_b_*: length of the beam; *E*: Young's modulus of the material; *G*: shear modulus of the material; *J_ξ_*: second order momentum of the beam cross-section around the principal axis that belongs to *x-y* plane; *J_p_*: second order polar momentum of the beam cross-section; *h*: thickness of the beam (measured along the *z*-axis); *b* depth of the beam cross-section (measured along the *x-y* plane).

### Load Condition A

**Figure 5.**
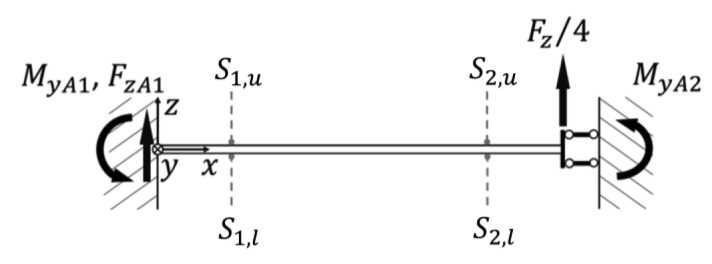
Load Condition A for beam-1 with force applied in the *z*-direction.

When a sensor is loaded with a force *F_z_* in the *z*-direction the force is equally divided on each beam with a load *F_z_*/4. Moreover the sensing-plate can only move by translation along the vertical direction. The scheme for the analytical solution can be represented as in Figure 4. The analytical solution for the strain on a generic point on the beam surface is given by:

(1) $${ɛ}_{xAi}={ɛ}_{xAi,u}=-{ɛ}_{xAi,l}=-\frac{F_{z}h}{8EJ_{\xi }}\left(x_{i}-\frac{l_{b}}{2}\right)$$

where *ε_xAi_* is the strain in the along *x*-direction at the point *S_i_* at a distance *x_i_* from the origin, that corresponds to the location of the sensitive point of the strain gauges as represented in Figure 5. Indexes *u* and *l* has been added to refer to upper or lower side of the beam.

### Load Condition B

**Figure 6.**
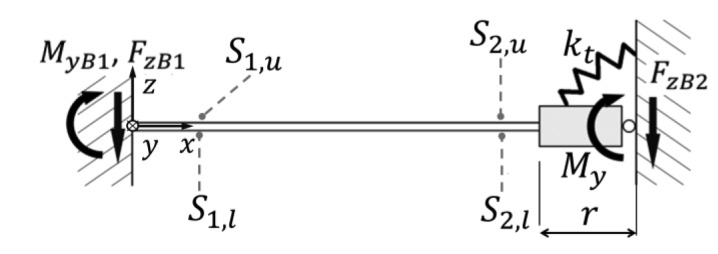
Load Condition B for beam-1 a torque *M* = *F_x_L* is applied along the *y*-axis is applied at *x* = *l*_b_.

The second possible load condition occurs when a force is exerted in the *x*-direction. In this case the system is geometrically symmetrical, but is loaded with anti-symmetrical loads thus the center of the sensing-plate is only allowed to rotate around the *y*-direction. In Figure 6 the loading condition with the hypothesis that load torque *M_y_* = *F_x_L* is equally divided between beam-1 and beam-3 is represented. Rotation of the angle *θ*_1_ can be computed as in [30], considering that the additional contribution to stiffness of the beam-2 and beam-4 that can be modeled as a torsion spring of stiffness *k_t_* = *l_b_*/(*GJ_p_*):

(2) $$\theta_{1}=\frac{\frac{F_{x}L}{2}}{\frac{12EJ_{\xi }r}{l_{b}^{3}}\left(r+\frac{l_{b}}{2}\right)+\frac{12EJ_{\xi }}{l_{b}^{2}}\left(\frac{r}{2}+\frac{l_{b}}{3}\right)+k_{t}}$$

(3) $${ɛ}_{xB1}={ɛ}_{xB1,u}=-{ɛ}_{xB1,l}=\frac{6h\theta_{1}}{l_{b}^{2}}\left[\left(r\left(\frac{x_{1}}{l_{b}}-\frac{1}{2}\right)+l_{b}\left(\frac{x_{1}}{2l_{b}}-\frac{1}{3}\right)\right)\right]$$

(4) $${ɛ}_{xB2}={ɛ}_{xB2,u}=-{ɛ}_{xB2,l}=\frac{6h\theta_{1}}{l_{b}^{2}}\left[\left(r\left(\frac{x_{2}}{l_{b}}-\frac{1}{2}\right)+l_{b}\left(\frac{x_{2}}{2l_{b}}-\frac{1}{3}\right)\right)\right]$$

Characteristic Matrix

The characteristic matrix can be written assuming the use of a full strain gauges Wheatstone-bridge for each beam:

(5) $$\tilde{v}=\left[\begin{array}{c} v_{1}\\ v_{2}\\ v_{3}\\ v_{4} \end{array}\right]=C\left[\begin{array}{c} p_{x}\\ p_{y}\\ p_{z} \end{array}\right]=C\tilde{p}$$

where *p_x_* = *F_x_/F_N_*, *p_y_* = *F_y_/F_N_*, *p_z_* = *F_Z_/F_N_* are the components of the force vector normalized respect to *F_N_*, that is the maximum nominal value, *ṽ* is the 4 × 1 vector of the voltage readings at the output of the four bridges of the sensor.

The connections are arranged this way: signals coming from strain gauges *S*_1_,*_u_ S*_2_,*_l_* are summed and the signals from strain gauges *S*_1_,*_l_* and *S*_2_,*_u_* are subtracted. It's worth noticing that, when using the full-bridge, the influence of the strain component generated by torsional deformation of the beams is theoretically cancelled. That is because the strain in the *x*-direction caused by torsional deformation at the point *S_i,u_* is equal and opposed to the strain at the point*S_i,l_*. According to the connection mentioned above the matrix *C* of Equation (5) can be written as follows:

(6) $$C=VG_{f}\left[\begin{array}{ccc} \frac{2{ɛ}_{xB1}-2{ɛ}_{xB2}}{F_{N}} & 0 & \frac{2{ɛ}_{xA1}-2{ɛ}_{xA2}}{F_{N}}\\ 0 & \frac{2{ɛ}_{xB1}-2{ɛ}_{xB2}}{F_{N}} & \frac{2{ɛ}_{xA1}-2{ɛ}_{xA2}}{F_{N}}\\ \frac{2{ɛ}_{xB1}-2{ɛ}_{xB2}}{F_{N}} & 0 & \frac{2{ɛ}_{xA1}-2{ɛ}_{xA2}}{F_{N}}\\ 0 & \frac{2{ɛ}_{xB1}-2{ɛ}_{xB2}}{F_{N}} & \frac{2{ɛ}_{xA1}-2{ɛ}_{xA2}}{F_{N}} \end{array}\right]=\left[\begin{array}{ccc} c_{1} & 0 & c_{2}\\ 0 & c_{1} & c_{2}\\ c_{1} & 0 & c_{2}\\ 0 & c_{1} & c_{2} \end{array}\right]$$

where *V* is the power supply voltage applied to the bridge and *G_f_* is the gauge factor of the employed strain gauges.

In practical use, the inverse problem is relevant to obtain the force components from the readings of the measured voltages. The inverse problem is redundant and can be inverted through least square method through the pseudo inverse matrix [31]:

(7) $$\left[\begin{array}{c} p_{x}\\ p_{y}\\ p_{z} \end{array}\right]=C^{+}\left[\begin{array}{c} v_{1}\\ v_{2}\\ v_{3}\\ v_{4} \end{array}\right]=\left[\begin{array}{cccc} -1/2c_{1} & 0 & 1/2c_{1} & 0\\ 0 & -1/2c_{1} & 0 & 1/2c_{1}\\ -c_{2}/4 & -c_{2}/4 & -c_{2}/4 & -c_{2}/4 \end{array}\right]\left[\begin{array}{c} v_{1}\\ v_{2}\\ v_{3}\\ v_{4} \end{array}\right]$$

In this particular case, the condition number of the characteristic matrix can be than expressed in a simple analytical form:

(8) $$\{\begin{array}{c} \text{CN}(C)=\frac{\sqrt{2}c_{1}}{2c_{2}}\;\text{if}\;c_{1}>c_{2}\\ \text{CN}(C)=\frac{2c_{2}}{\sqrt{2}c_{1}}\;\text{if}\;c_{2}>c_{1} \end{array}$$

It is interesting to note that the system is intrinsically isotropic along the *x-y* direction. Such a characteristic is particularly interesting since in the in hand exoskeletons two different type of forces are delivered to the user finger: normal and tangential. Accordingly, the spring has the potential property of showing intrinsic isotropy towards the tangential forces (*F_x_* and *F_y_*) and a different sensitivity toward normal forces (*F_Z_*). The proof of such property can be given considering the minor obtained extracting the first two rows of the matrix *C*^+^ and verifying that the condition number is always equal to the unit.

## 4. Overload Protection

Overload protection is a mechanism that protects the sensing spring of force sensors against functional failure caused by forces that surpass the maximum nominal limits. This means that the spring is not only protected against breaking, but also against permanent deformations. Usually overload protection is designed by introducing unilateral constraints that limit the deformation of the spring through some limitation on the displacement of the sensing-plate of the sensor. A perfect set of constraints that implements an overload protection has to permit the free deformation of the spring when the applied force is lower than the admissible threshold and has to operate only when the force exceeds such limits.

The design of an efficient overload protection system is not always a simple procedure in multiple DoF force sensors. The problem requires choosing two sets of surfaces (constraint-surfaces), fixed respectively to the base of the sensor and to the sensing-plate, that come into contact when the force reaches the maximum limits. Once the contact takes place the load condition changes with the effect of partially or completely relieving the spring.

In the next section the authors propose a solution for overload protection which adapts optimally to the proposed sensor spring. The study of such protection goes through the analysis of the rigid body motion of the sensing-plate of the force sensor and through the choice of a set of constraint-surfaces that properly limit its displacements.

### 4.1. Analysis of Displacements

The shaping of the constraint-surfaces that guarantee an optimal matching between the allowed and the needed displacements of the sensing-plate has to go through a rigid body motion analysis of the displacements. Due to the high stiffness of the spring against forces that lay on the *x-y* plane and against torque in the *z*-direction (with reference to Figure 3), it is possible to assume that the motion of the sensing-plate is restricted to rotations around the *x*- and *y*-axis and translation along the *z*-axis.

Moreover, thanks to the isotropy of the stiffness matrix in the *x-y* plane the problem comes down to a planar motion analysis. Under these assumptions it is possible to describe completely the displacement of the sensing-plate with a two-dimensional vector δx^*^ = [*δθ_x_ δz*]*^T^*, indicating with the introduced components the rotation around the *x*-axis and the translation in the *z*-direction. The corresponding stiffness matrix can be written as follows:

(9) $$\delta x^{*}=K^{-1}F^{*}=\left[\begin{array}{cc} \frac{1}{k_{\theta }} & 0\\ 0 & \frac{1}{k_{z}} \end{array}\right]\;\left[\begin{array}{c} F_{x}\\ F_{z} \end{array}\right]$$

In order to select eligible constraint-surfaces, it is useful to analyze the motion of different points that belong to the sensing-plate when a unit force is exerted on the sensing-point of the sensor. The displacement $\delta p_{i}^{*}$ of a point $p_{i}^{*}$ that moves with the sensing-plate is given by the linear relation that considers small displacement hypothesis:

(10) $$\delta p_{i}^{*}=J_{pi}\delta x^{*}=J_{pi}KF^{*}=DF^{*}$$

where *J_pi_* is the Jacobian matrix whose column are the displacement vectors of the point $p_{i}^{*}$ when unitary displacements are imposed for *δθ_x_* and *δz*.

**Figure 7.**
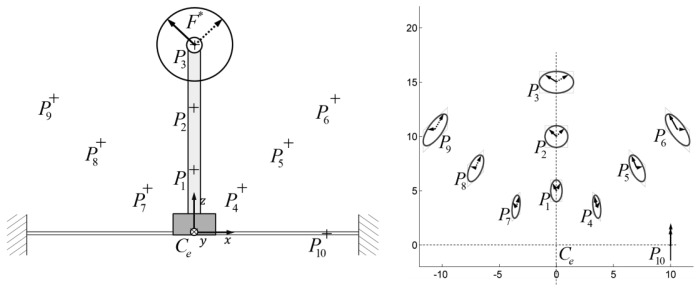
Scheme of the field of displacement (on the right) of points that move with the sensing-spring when the magnitude of applied force reaches the boundary of the nominal values (‖F^*^‖ = *F_N_*).

It is useful to draw and visualize the field of displacement that the sensing-plate assumes as the force varies in the nominal force range (see Figure 7 on the right):

(11) $$\left\|F^{*}\right\|\leq F_{N}$$

where F^*^ is the two-dimensional vector of forces in the *x*- and *y*- directions.

The displacement of a point $p_{i}^{*}$ produced by the application of constant-magnitude forces can be calculated as in [32]. The locus of the points described by $p_{i}^{*}$ is an ellipsis whose main axis are aligned with the Eigen-vector of the compliance matrix *D*. In Figure 7 the displacements are plotted for *k_θ_* = 200 *N/rad*, *k_z_* = 20 *N/mm*, ‖*F*‖= 1 *N*.

One possible way to identify a surface that minimally satisfies the required displacements is to choose any wanted surface (*S_p_*) that belongs to the sensing-plate and to draw the envelope of the ellipsis of maximum displacements of each point of the chosen surface. The fixed constraint-surface that minimally guarantees the necessary displacements will be that envelope (*S_e_*).

**Figure 8.**
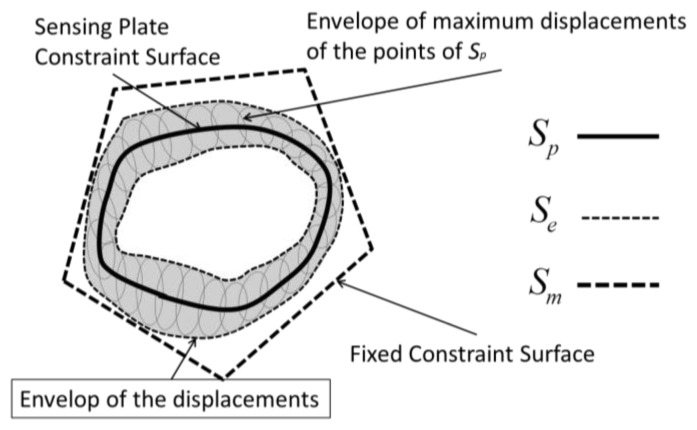
Scheme of the principle for the synthesis of candidate surfaces for overload protection.

A trade-off that has to be accepted is due to manufacturing problems of such general surfaces, thus the ideal surface is approximated with a surface that can be reasonably manufactured (*S_m_*). A graphical representation of the problem is shown in Figure 8.

### 4.2. Solution for the Overload Protection

**Figure 9.**
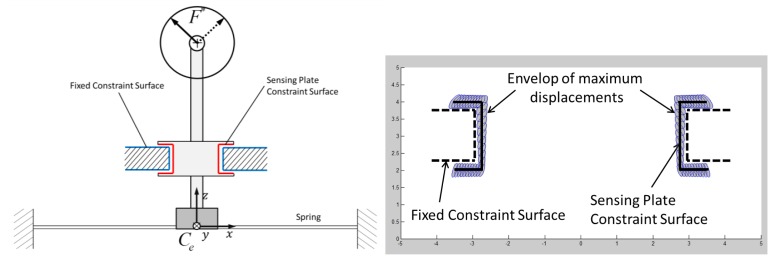
Scheme of the working principle for the adopted overload protection mechanism. The overload protection is located between the spring and the sensing-point. On the right side it is shown how the fixed constraint-surface was chosen to comply with the displacement envelope.

The design choice for the overload protection followed the procedure explained in the previous section and started from the choice of a constraint-surface belonging to the sensing plate (*S_p_*). The shape of the surface is shown in Figure 9(left). The envelope of the maximum displacements of the points that belongs to this surface is represented in Figure 9(right). The envelope has global toroidal shape. The internal part of the toroid has a conical surface that can be well approximated with a cylindrical surface, depending on the dimensions of the device.

**Figure 10.**
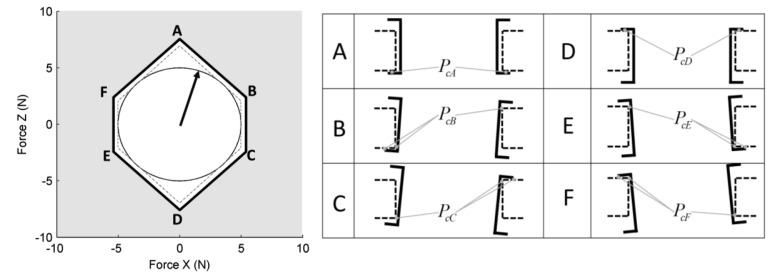
Representation of the overload protection limits to the applied force. Overload protection is active for the forces that fall in the grey area (left picture) outside the polygon ABCDEF. It's important to underline that nominal forces fall inside the circle that is in turn fully included in the area of the polygon. The figure on the right shows the actual displacements of the moving constraint-surface produced by forces that corresponds to the points ABCDEF.

A cylindrical surface has been chosen and manufactured for the prototype. Such an approximation introduces the possibility of overcoming the nominal force before the overload protection can operate. In Figure 10 a graphical representation of the resulted constraints on the applied forces are shown. The nominal force is included in a circular shape while the overload constraint has a polygonal shape that includes the nominal force circle. The limit conditions for the displacements of the moving constraint surface produced by the application of forces that correspond to the vertex of the polygon are represented in Figure 10(right).

## 5. Prototype Design and Experimental Characterization

### 5.1. Prototype Design

Two prototypes of the proposed sensor have been designed and developed with the final goal of integrating it with the dual finger hand exoskeleton described in [18]. This device can independently exert a 3-DoF force in the range of ±5 N on both index and thumb fingers. The sensor's spring and the electronics have been dimensioned aiming at the same measuring range. In this section, the authors briefly describe the mechanical dimensioning of the sensor spring and the performance verification through a FEM analysis of the sensor and present the custom integrated conditioning electronics that has been designed. Lastly, the developed prototypes are experimentally characterized and tested.

#### 5.1.1. Mechanical Dimensioning

The main objective of the mechanical dimensioning of the proposed force sensor is to maximize the resolution over the three directions of sensing. The optimal resolution is obtained by maximizing the sensitivity of the sensor voltage output with respect to the applied force. Therefore the following guidelines and solutions have been considered:

- optimize the condition number of the characteristic matrix weighted with respect to the maximum forces along the different directions;
- use a full gauges bridge for each single beam in order to have a summation of strain contributions and temperature compensation;
- employ specific conditioning and acquisition electronics, that will be described in the next section, in order to reduce the noise that affects the analogue signals.

The design started from the hypothesis of fixing the encumbrance of the force sensor in order for the maximum device encumbrance to be included in a square of 25 mm.

High yield strength 301 spring steel with maximum yield strength of *σ_vm_*= 930 MPa was used for the manufacturing of the sensor spring. A cautionary factor of safety (*FoS* = 2.5) has been chosen for considering possible stress concentration at the base of the beams and tolerances of the overload protection.

With the given constraints and using the analytical formulation described in Section 3 we define the following dimension (with the symbols used in Section 3.1): *l_b_* = 7.4 mm; *E* = 200 GPa; *h* = 0.4 mm; *b* = 3 mm.

Special care has been dedicated to the definition of engineering tolerances and manufacturing of the spring. Manufacturing precision is in fact an important issue for force sensors design and it is particularly relevant in this case where mechanical components of small dimensions are to be employed. The spring was manufactured through a high precision CNC milling cutter that allows to achieve high grade precision (IT Grade 6) for the relevant dimensions. High resistance (*R* = 5 kΩ) Vishay N3K series strain gauges made of Karma-alloy (G_f_ = 2.1) were chosen for their small dimensions and for their possibility to be powered by means of a standard power supply voltage of ±5–10 V.

#### 5.1.2. FEM Verification

**Figure 11.**
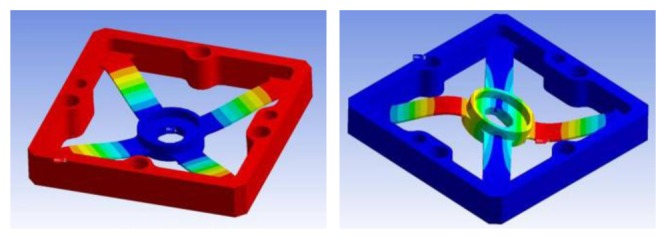
Screen-shot of the displacement field resulting from the FEM analysis for the nominal force in Load Condition A (**left**) and Load Condition B (**right**). Displacements are amplified by a factor of 10 for the purposes of the visualization.

A FEM model of the spring of the sensor has been developed according to the geometrical dimensions that have been defined in the previous section (see Figure 11). The FEM model has been employed for two types of verification. First, the analytical estimation of the characteristic matrix of Equation (6) has been verified. A difference between the two models up to 10.2% was recorded; in particular, higher values for the analytical estimation for each element of the characteristic matrix were observed. This difference can be attributable to a “higher compliance” of the analytical model due to approximations that are taken with the slender-beam assumption: (1) the ratio between thickness and height of the cross-section dimensions is not close to the unit (it is approximately 9); (2) the end-attachment is not clamped to a straight wall as assumed in the analytical model, but rather it is clamped to the corner formed by two walls reducing the effective length of the beam; (3) in the analytical model the beam-ends are clamped to a rigid wall while in the FEM model the beam-ends are clamped to a frame that is anchored with four screws introducing an additional compliance to the structure. Assumptions (1) and (2) have a major influence and tend to increase the global stiffness of the real spring. As consequence a lower sensitivity of the spring is observed. Factor (3) is less relevant since the frame has been designed to minimally deform under applied loads.

A second verification that has been conducted is related to the structural verification of the spring in the extreme case of solicitation of overload described in Figure 10. A displacement has been imposed to the sensing-plate in order to reach each of the vertex of the polygon ABCDEF. For each single position the maximum equivalent Von-Misess stress has been evaluated. An updated factor of safety FoS^*^ = 1.8 has been evaluated.

### 5.2. Conditioning and Acquisition Electronics

**Figure 12.**
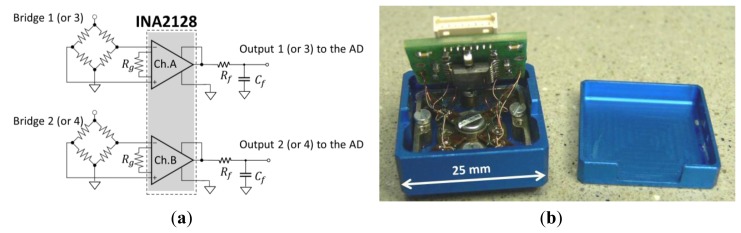
Scheme of the one half of the signal conditioning electronic circuit with *R_g_*: gain regulation resistance; *R_f_* and *C_f_*: RC low pass filter components (**a**); picture of the force sensor assembly with the integrated electronics, raised to show the strain gauges cabling (**b**).

A custom on-board conditioning electronics has been developed using the Instrumentation Operational Amplifier INA2128 by Texas Instruments^®^. This is a double channel voltage amplifier with a variable gain in the range of 100–1,000 settable through an external resistor. A pair of INA2128 units have been employed to implement the four required channels. The output signals are filtered through an on board Low-Pass Filter (LPF), set to a cut-off frequency of 500 Hz. The variable gain of the amplifier was set to 250 to provide an output in the range of ±5.5 V. The adopted scheme is represented in Figure 12(a). The layout of the PCB was optimized and miniaturized for the integration into the body of the force sensor [see Figure 12(b)].

### 5.3. Experimental Characterization

**Figure 13.**
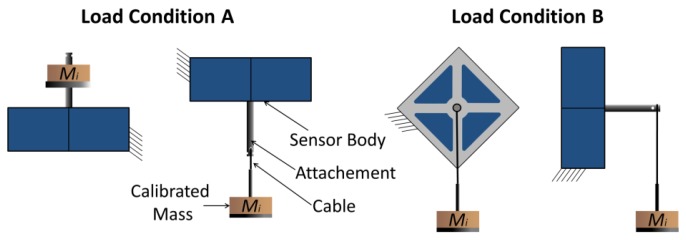
Scheme of the characterization procedure for the two different load conditions.

**Figure 14.**
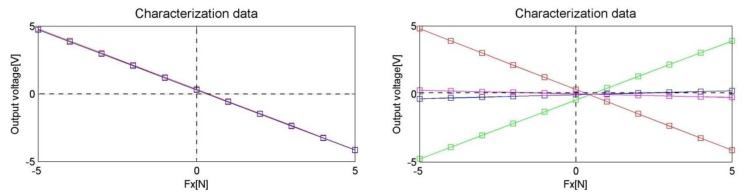
Plot of the measures of one of the four signals (***v*_11_**) during loading (blue) and unloading cycles (red) and plot of averages between unloading and loading values for all the four signals. Force is applied along x-axis.

Experimental characterization of the two manufactured prototypes has been conducted and results were compared with the estimations provided by the analytical and numerical (FEM) models. The measurements were conducted arranging the sensor in two different positions that corresponded to Load Condition A and Load Condition B (see Figure 13). The sensor has been loaded along the *x-y* and *z* directions using 11 different loads (five different masses plus no-load) vertically attached at the sensing-point. Two series of measures have been acquired corresponding to loading and unloading cycles using a high precision voltmeter (Tektronix DM512). Each single measuring cycle has been repeated 3 times showing a maximum repeatability error lower than 2 mN (0.04% of rated load). In Figure 14 the plot of the loading and unloading curves is shown for a loading cycle along the *x*-axis. The plotted values are averaged over three measurements cycles. Static offsets have been measured using the same experimental set up with no load applied.

Experimental characteristic matrixes (see Table 1) have been computed for the two sensor-prototypes with linear least square fitting of values averaged between the loading and unloading data.

Measured offsets are most probably attributable to residual stress caused by the heated-gluing process of strain gauges. Voltage offsets are constant over time and they can be compensated, however their value should be minimized to avoid limitations on the sensing range of the sensor.

Using the characteristic matrixes shown in Table 1 without further compensations it is possible to evaluate the main parameters of the sensing system. In Table 2 the main specifications for the sensor S1 are reported as reference.

## 6. Integration and Tests

### 6.1. Integration on Dual Finger Hand Exoskeleton

**Figure 15.**
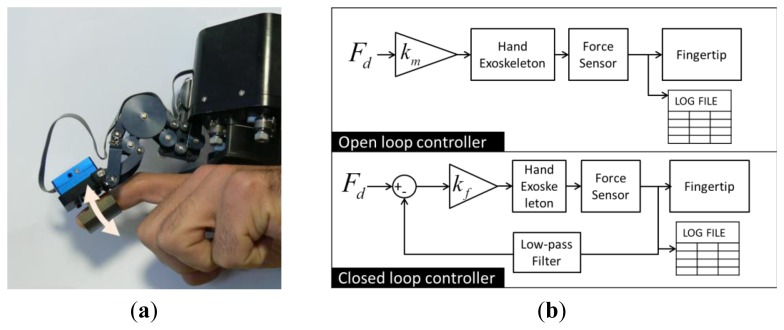
Picture of the experimental setup. (**a**) The finger of the 3-DoF hand exoskeleton is connected to a grounded reference plate. (**b**) Scheme of the two controllers that are compared.

The sensors described in the previous section have been integrated on a hand exoskeleton haptic device (see Figure 15). It is a dual finger hand exoskeleton capable of exerting forces on index and thumb fingers [18]. The device is composed by two independent 3-DoF fingers able to exert controlled forces in the range of ±5 N along any desired direction.

### 6.2. Experimental Test

With the aim of quantitatively verifying the performance improvement that can be achieved with the novel sensor, a preliminary test has been conducted. The scope of such a test was not to provide an exact estimation of the maximum performances that can be achieved, but rather to provide an approximated quantitative measure of the improvement in terms of capability of compensation of disturbance forces. An in-depth investigation of the optimization of control and relative performances is foreseen, but it is beyond the scope of this work.

Tests have been conducted using one finger of the hand exoskeleton and the sensor S1 (see Table 1). The proximal link of the hand exoskeleton, *i.e.*, the dorsal plate, has been grounded in order to avoid possible disturbances introduced by unwanted displacement of the hand.

The test consisted in performing a cyclical task of flexing the index finger in a natural way starting with the finger in a relaxed extended position [see Figure 15(a)]. The testers were asked to flex the index finger and invert the motion as soon as the fingertip has rotated approximately of 30°–40°. No particular measures have been taken for controlling the accuracy of the rotation as its magnitude was not relevant. The testers were asked to repeat cyclically the movement with a frequency of approximately 0.5 Hz. An audio pulsating signal was presented during the test to help to keep the frequency of the movement cycle roughly constant.

Two basic control algorithms were compared. The first one [see Figure 15(b)] is an open loop controller that does not include the force measured in the control loop, but employs the sensor only for measuring the contact force at the fingertip of the user. The second control algorithm is a simple closed-loop force controller with force compensation loop. The feedback signal from the force sensor is filtered through a LPC with cut-off frequency set at 25 Hz. The gain *k_f_*, in the second control scheme, regulates the amount of compensation improving the performance of the system but it is limited by stability issues. The gain has been increased and set up to 90% of the stability limit. Advanced types of controller including PID, Kalman filters, or position/velocity-based force controller will be studied for further improving the global performances but this is beyond the scope of this work.

### 6.3. Results

**Figure 16.**
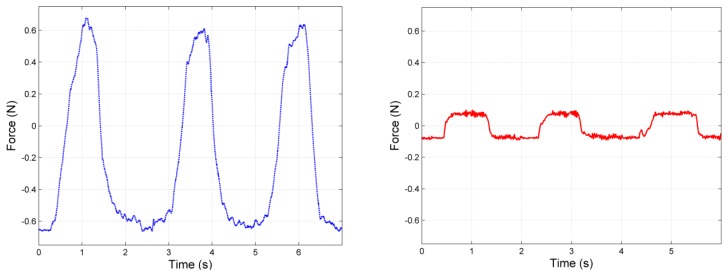
Comparison between the residual uncompensated force for the open loop controller (**left**) and closed loop controller (**right**) for the described test (*F_d_* = 0).

The two control algorithms presented in the previous section were compared commanding a null desired force (*F_d_* = 0) and measuring the residual uncompensated force that is delivered to the fingertip. A plot of the results is represented in Figure 16 showing the magnitude of the residual uncompensated forces in the case of open loop (left) and closed loop (right) controller.

Average values for the compensation on five cycles during three different test sessions show an improvement of force-error reduction of approximately 87%. Further improvements are foreseen through the implementation of advanced control algorithms.

## 7. Conclusions

This study has endeavored to design and test a novel three-axis force sensor. The sensor has been purposely developed for sensing forces delivered to fingertips by a double finger three degrees of freedom haptic interface. The aim of the system is to artificially generate forces on the user fingertips for simulating the physical interactions that occur during Precision Grasping and Surface Exploration with bare fingers. Such an objective is extremely challenging since the forces involved in the interaction with bare finger range from tenths of milli-Newtons up to tens of Newtons. For this reason a high performance force sensor is highly desirable, especially for the improvement of force resolution. The design of the force sensor has been conducted with the goal of maximizing force resolution through a proper choice of the spring structure and the choice of low-noise integrated electronics. Special care has been dedicated to guarantee reliability of the sensor introducing a solution for its overload protection and a specific method for its design.

Tests have been conducted on a dual finger hand exoskeleton showing the capabilities of the sensor to be effectively employed for improving performances of haptic interfaces that lack of force sensing. An average improvement of 87% on force disturb during free motion has been recorded. These estimations are subject to be further improvement with an in-depth study on an optimized controller.

## Figures and Tables

**Table 1.** Characteristic Matrix of the two developed sensor prototypes.

**S1**	***px*_1_ [V]**	***py*_1_ [V]**	***pz*_1_ [V]**	**Off-Set [V]**
*v*_11_	−0.891	0.082	0.200	0.312
*v*_21_	0.057	−0.845	0.191	−0.015
*v*_31_	0.865	0.061	0.188	−0.411
*v*_41_	−0.052	0.854	0.210	0.002
**S2**	***px*_2_ [V]**	***py*_2_ [V]**	***pz*_2_ [V]**	**Off-Set [V]**

*v*_12_	−0.821	0.078	0.194	−0.721
*v*_22_	−0.012	−0.797	0.182	0.423
*v*_32_	0.812	0.091	0.179	−0.021
*v*_42_	−0.065	0.801	0.190	0.034

**Table 2.** Main performance parameters for the developed sensor S1.

**Parameter**	**Value**	**Unit**	**% of RL**
Measuring Range	5	N	NA
Linearity	2.7	mN	0.06%
Hysteresis	10	mN	0.21%
Cross talk	5.0	mN	0.1%
Resolution *	2.0	mN	0.04%

* Resolution is mainly limited by noise; for characterization, a high precision low noise power supply has been employed which shows peak to peak noise *V_p_*_−_*_p_* ≤ 0.3 *mV*.
